# Supplementary material for: Machine Learning Quantification of Intraepithelial Tumor-Infiltrating Lymphocytes as a Significant Prognostic Factor in High-Grade Serous Ovarian Carcinomas
Source: Int J Mol Sci. 2023 Nov 7;24(22):16060. doi: 10.3390/ijms242216060 (PMC10671555; doi:10.3390/ijms242216060)
Supplement: Supplementary file 1 [file ijms-24-16060-s001.zip › ijms-2685419-supplementary.pdf]

Supplementary Table S1. Comparative Analysis of Overall Survival and Platinum-Free Interval Stratified by intraepithelial TILs and stromal TILs in Different Patient Subgroups.

				**Overall survival**			**Platinum-Free Interval**		
				% survivors (median)	*p*	HR (95% CI)	Median	*p*	HR (95% CI)
Neoadjuvant therapy	YES (32)	ieTILs	High	48.1% (46 m)	*p*=0.09	2.08 (0.86-5.06); *p*=0.1	13 m	*p*=0.13	1.76 (0.83-3.73); *p*=0.1
			Low	7.14% (37 m)			11 m		
		sTILs	High	27.4% (46 m)	*p*=0.4	1.45 (0.66-4.88); *p*=0.5	13 m	*p*=0.56	1.25 (0.58-2.67); *p*=0.6
			Low	12.8% (45 m)			11 m		
	NO (44)	ieTILs	High	58.8%	*p*=0.06	2.93 (0.92-9.37); *p*=0.07	48 m	***p*=0.0009**	**3.45** (1.60-7.45); *p*=0.002
			Low	23.5% (70 m)			15 m		
		sTILs	High	63%	***p*=0.02**	**4.38** (1.22-15.75); *p*=0.02	29 m	***p*=0.009**	**2.68** (1.22-5.91); *p*=0.01
			Low	21.2% (70 m)			15 m		
Somatic *BRCA*	Mutated (24)	ieTILs	High	68.8%	***p*=0.02**	**4.16** (1.09-15.88); *p*=0.04	26 m	***p*=0.04**	**2.72** (1.01-7.34); *p*=0.05
			Low	0% *(53 m)*			12 m		
		sTILs	High	53.6%	*p*=0.4	1.75 (0.53-5.77); *p*=0.4	26 m	***p*=0.02**	**3.08** (1.37-6.92); *p*=0.03
			Low	17.5% (53 m)			12 m		
	Non-mutated (52)	ieTILs	High	45.2% (80 m)	*p*=0.1	1.85 (0.81-4.52); *p*=0.1	29 m	***p*=0.004**	**2.38** (1.33-4.53); *p*=0.006
			Low	17.7% (46 m)			16 m		
		sTILs	High	55.9%	*p*=0.07	2.10 (0.92-4.82); *p*=0.08	27 m	*p*=0.25	1.42 (0.76-2.64); *p*=0.3
			Low	22% (46 m)			17 m		
Complete cytorreduction	YES (48)	ieTILs	High	61.8%	*p*=**0.02**	**3.05** (1.07-8.71); *p*=0.04	36 m	***p*=0.004**	**2.99** (1.44-6.21); *p*=0.003
			Low	0% (57 m)			18 m		
		sTILs	High	47.5% (80 m)	*p*=0.2	1.78 (0.68-4.63); *p*=0.2	29 m	*p*=0.4	1.41 (0.70-2.86); *p*=0.3
			Low	0% (63 m)			15 m		
	NO (28)	ieTILs	High	34.9% (52 m)	*p*=0.1	2.174 (0.8327-5.675); *p*=0.1	19 m	***p*=0.03**	**2.49** (1.04-5.94); *p*=0.04
			Low	15.4% (36 m)			11 m		
		sTILs	High	38.9% (52 m)	*p*=0.5	1.477 (0.5229-4.171); *p*=0.5	19 m	*p*=0.1	2.03 (0.81-5.05); *p*=0.1
			Low	20.7% (36 m)			12 m		

HR: Hazard Ratio; ieTILs: intraepithelial tumor-infiltrating lymphocytes; sTILs: stromal tumor-infiltrating lymphocytes; m: months; CI: confidence interval.

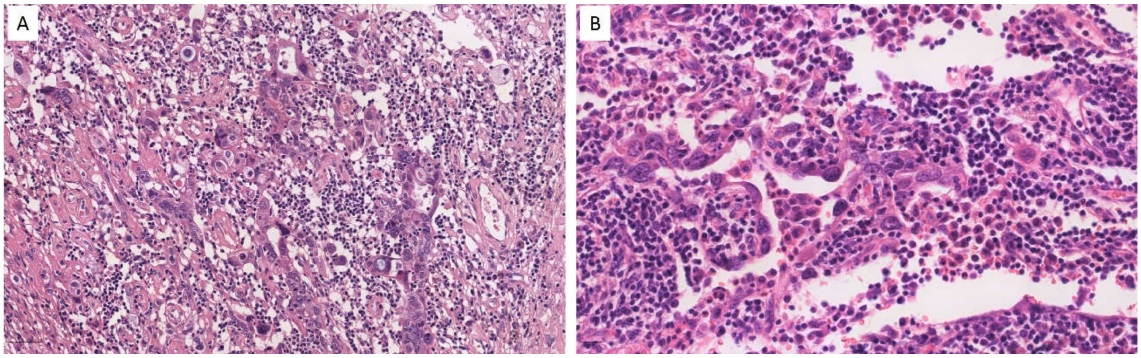

Supplementary Figure S1. Two examples of carcinoma with intense infiltration by immune cellularity, with *tumor cord colonization* (A. HE; 40×. B. HE; 200×).

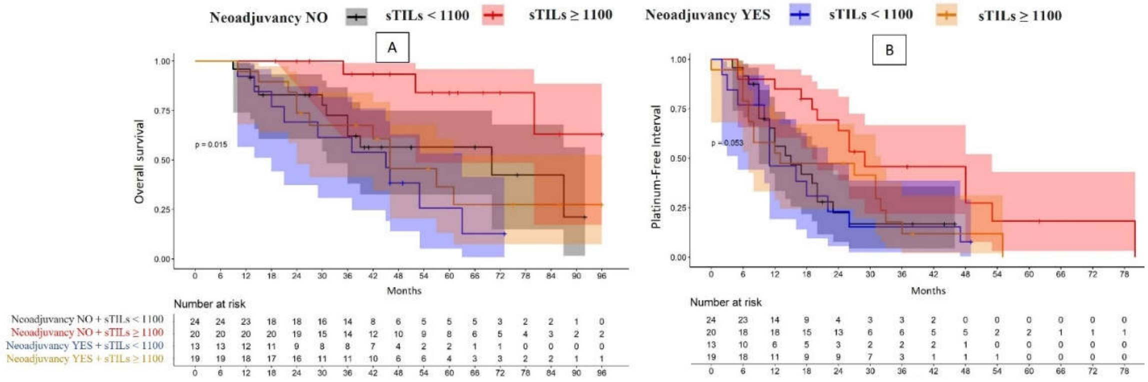

Supplementary Figure S2. A-B: Comparative study of overall survival and platinum-free interval in patients with high-grade serous ovarian carcinoma based on whether they received neoadjuvant treatment and sTIL concentration (A and B, respectively).

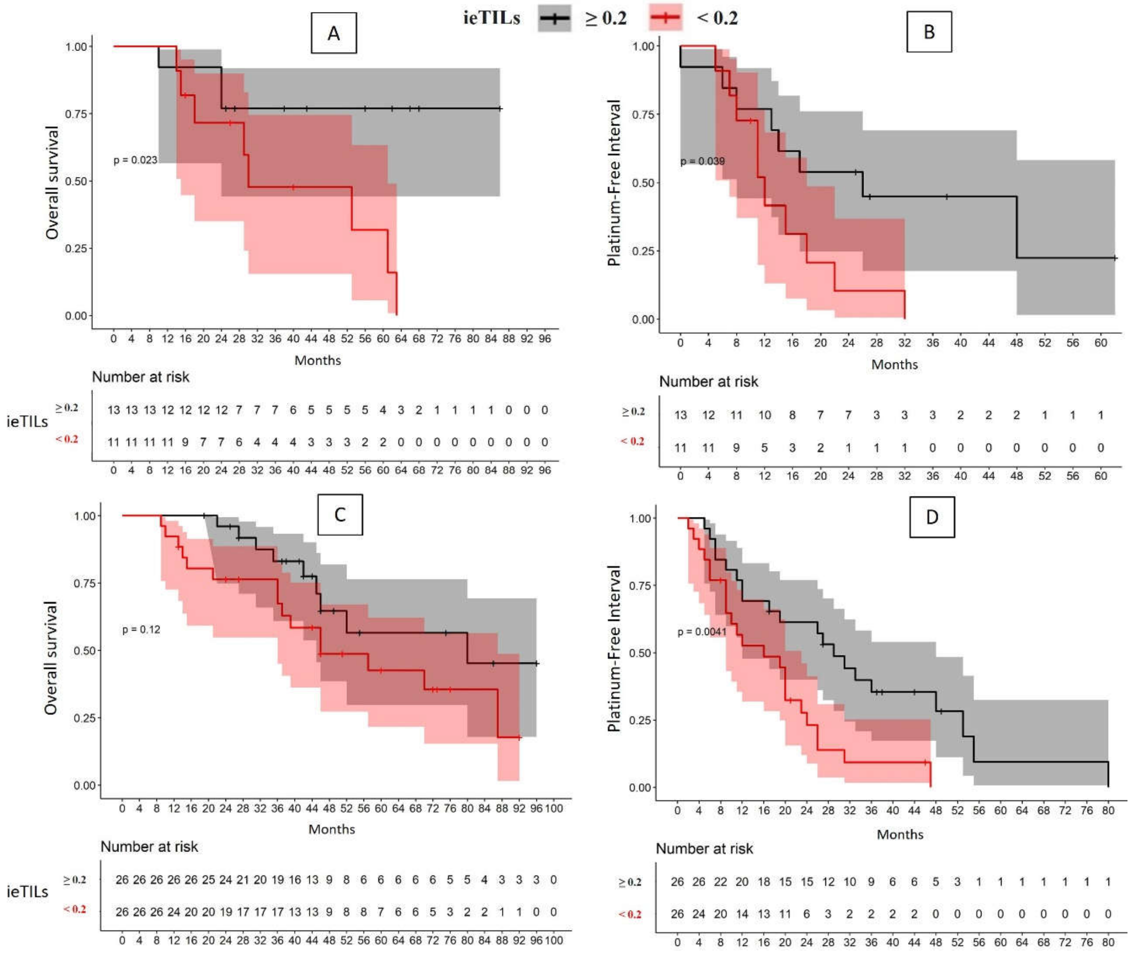

Supplementary Figure S3. A-D: Comparative curves of overall survival and platinum-free interval of patients with high-grade serous ovarian carcinoma stratified by ieTILs in patients with BRCA mutation (A and B, respectively), and without BRCA mutation (C and D, respectively).

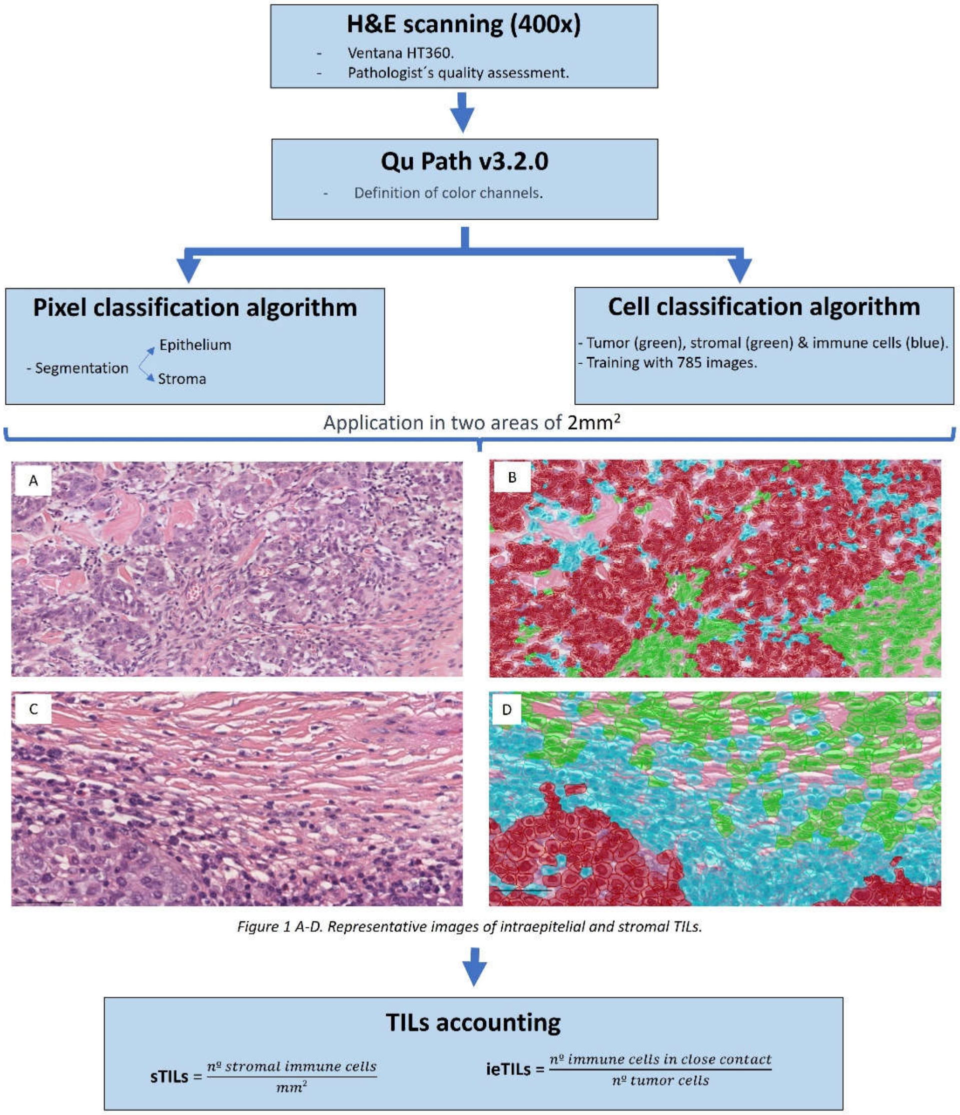

Supplementary Figure S4. Flow diagram representing the training process for the digital analysis program and tumor-infiltrating lymphocyte quantification system categorized by location.
